# Influence of exercise self-efficacy on physical activity among psychologically distressed patients undergoing cardiac rehabilitation: secondary data analysis of a randomized controlled trial

**DOI:** 10.1186/s40359-026-04004-8

**Published:** 2026-02-03

**Authors:** Viktoria Liv Pollok, Friederike Thome-Soós, Dieter Benninghoven, Matthias Bethge

**Affiliations:** 1 Institute for Social Medicine and Epidemiology, University of Lübeck, Ratzeburger Allee 160, 23562 Lübeck, Germany; 2 Mühlenbergklinik Holsteinische Schweiz, Frahmsallee 1-7, 23714 Bad Malente, Germany

**Keywords:** Cardiac rehabilitation, Exercise self-efficacy, Physical activity, Behavior change, Psychological comorbidity, Cardiovascular diseases

## Abstract

**Background:**

Cardiac rehabilitation programs are commonly employed to address modifiable lifestyle factors, such as physical inactivity. However, adherence to exercise recommendations following these interventions remains suboptimal. Psychological burdens can further impede patients’ ability to exercise.

**Aim:**

The aim of this study was to investigate the role of exercise self-efficacy (ESE) in influencing physical activity among psychologically distressed patients in cardiac rehabilitation.

**Methods:**

Data from a randomized controlled trial involving 305 participants who completed a four-week cardiac rehabilitation program were analyzed. ESE was measured using the Exercise Self-Efficacy Scale, while physical activity was assessed by aggregating the duration and frequency of up to three weekly activity types participants engaged in. Correlation analyses were conducted to examine the relationships between ESE and potential explanatory variables. A linear regression model was employed to assess the predictive role of baseline ESE on physical activity at the 3-month follow-up. Changes in ESE and physical activity over time were analyzed using paired t-tests and a mixed-effects model.

**Results:**

At the start of rehabilitation, weekly physical activity averaged 108 min, while ESE reached a mean of 28.2 points. Baseline ESE significantly predicted physical activity at the 3-month follow-up when controlling for baseline activity (b = 0.10, *p* = 0.006). T-tests showed a 1.75-point increase in ESE (*p* < 0.001) and a 68.23-minute increase in physical activity (*p* < 0.001) over time. Mixed-effects regression analysis indicated that each one-point increase in ESE was associated with a 12.88-minute increase in physical activity at follow-up (*p* < 0.001).

**Conclusion:**

ESE may have a considerable impact on physical activity among psychologically distressed patients in cardiac rehabilitation and should therefore be considered as a relevant outcome parameter when designing new intervention components. For clinical practice, these findings support the integration of routine screening for ESE upon rehabilitation entry as well as the incorporation of targeted strategies to enhance self-efficacy within cardiac rehabilitation programs.

**Trial registration:**

German Clinical Trials Register (DRKS00029295, June 21, 2022).

**Supplementary Information:**

The online version contains supplementary material available at 10.1186/s40359-026-04004-8.

## Introduction

For several decades, cardiovascular diseases (CVD) have consistently remained the primary cause of morbidity and mortality worldwide [1]. In 2021, CVD accounted for five of the top ten causes of mortality in Germany, representing 48.1% of all fatalities [2]. In the German population, overall lifetime prevalence of significant CVD reached 12.0% in 2021, with men exhibiting a higher prevalence (13.3%) compared to women (10.7%). This prevalence notably increases with age, reaching 45% among individuals aged 80 years and older [3]. Consequently, CVD represent the largest share of healthcare expenditures, accounting for 13.7% of the total disease-related costs in Germany [4]. Besides environmental and metabolic factors, behavioral contributors such as physical inactivity are well-established risk factors for CVD [5]. Accordingly, a study by Tang et al. (2013) revealed that only 17% of patients with self-reported coronary heart disease perform the recommended levels of physical activity [6].

In 2021, low physical activity was estimated to be attributable to 0.397 million deaths and 7.2 million disability-adjusted life years [5]. Conversely, achieving sufficient activity levels can yield substantial benefits even after the onset of CVD. A cohort study with 441.798 participants found that increasing physical activity by 500 MET-minutes per week reduced the mortality risk by 14% in individuals with established CVD compared to a 7% reduction in those without CVD. Additionally, individuals with CVD who maintained high levels of physical activity (≥ 1000 MET-minutes per week) had mortality risks that were comparable to or lower than those of individuals without CVD [7]. In support of these findings, a systematic review found that exercise-based rehabilitation post-myocardial infarction significantly reduced the risk of a second cardiac event, as well as cardiac and all-cause mortality, which further emphasizes the critical role of physical activity in improving outcomes for patients with established CVD [8].

The adjustment of modifiable risk factors such as low physical activity is a pivotal objective in cardiac rehabilitation [9]. With structured exercise promotion as a central component, cardiac rehabilitation has shown significant improvements in both clinical-prognostic outcomes and participation goals among this population [10–12]. However, adherence to exercise recommendations post-rehabilitation is low, and many patients fail to maintain the health benefits achieved during these programs. A systematic review found that only half of the trials included successfully enhanced activity behaviors following exercise-based cardiac rehabilitation [13]. Another review reported activity improvements in a mere 26% of participants [14]. Similarly, a randomized controlled trial found that 46.4% of cardiac rehabilitation participants did not adhere to physical activity recommendations in the long term after completing the program [15].

One reason for this phenomenon may be that exercise interventions typically focus solely on physical capabilities, neglecting the social-cognitive factors essential for sustained exercise adoption and maintenance [16]. In addition, psychological illnesses often occur simultaneously among patients with CVD. For instance, the prevalence of major depression in patients with coronary heart disease has been reported to be as high as 40% [17]. Depression is associated with poorer disease outcomes, reduced functional capacity, and increased long-term mortality in patients with CVD [18], which further complicates efforts to achieve successful exercise modification within this population. Importantly, these burdens can also impair self-regulatory behaviors by reducing energy and concentration underscoring the need to identify factors that facilitate exercise engagement in this vulnerable population to better address and overcome these barriers.

Self-efficacy, defined as the belief in one’s capabilities to organize and execute the necessary actions to achieve a desired outcome despite barriers, is a well-established construct in behavior change research [19]. Introduced by Albert Bandura in the 1970s, Social Cognitive Theory (SCT) provides a widely used framework for explaining behavior, with self-efficacy as a central mechanism. A systematic review has confirmed SCT as a useful framework for understanding physical activity behavior [20]. Exercise self-efficacy (ESE), the domain-specific form of self-efficacy, has been shown to be positively associated with physical activity, both during and after cardiac rehabilitation [21].

In this context, this study aims to investigate whether ESE plays a role in behavior change in psychologically distressed patients undergoing cardiac rehabilitation, addressing the following research questions:

1. What health- and work-related, psychological, and sociodemographic factors are associated with ESE?
2. Does ESE at the beginning of rehabilitation predict the level of physical activity at the 3-month follow-up?
3. Do ESE levels change significantly between the start and the end of rehabilitation?
4. Does physical activity change significantly between the beginning of rehabilitation and the 3-month follow-up?
5. Do changes in ESE between the initiation and completion of rehabilitation correspond to alterations in physical activity between baseline and the 3-month follow-up?

## Methods

### Study design

The present cohort study investigating the association between ESE and physical activity is based on a secondary analysis of data derived from a randomized controlled trial originally conducted to assess the effectiveness of a cognitive-behavioral rehabilitation program compared to standard care [22]. The program tested in this trial is a four-week multimodal intervention incorporating psychological and exercise components beyond standard rehabilitation programs. It aims to determine if participants in cognitive-behavioral rehabilitation achieve more favorable outcomes compared to those in standard care, with the primary outcome being cardiac anxiety 12 months post-intervention. The intervention included structured cognitive-behavioral therapy sessions and additional supervised exercise, all delivered within the rehabilitation center. Compared to traditional cardiac rehabilitation, it places greater emphasis on psychological support and behavioral strategies to improve exercise adherence and lifestyle modifications. More information can be found elsewhere [22].

Reporting in this paper adhered to the STROBE Statement (Strengthening the Reporting of Observational Studies in Epidemiology) [23]. The Ethics Committee of the University of Lübeck confirmed compliance with ethical standards for the CBR-CARDIO trial on May 11, 2022 (22–160). The original trail was registered in the German Clinical Trials Register (DRKS00032536).

### Setting

Recruitment was conducted at a rehabilitation facility in northern Germany (https://www.muehlenbergklinik-holsteinische-schweiz.de) from June 28, 2022, to January 5, 2024. After informed consent, patients were randomly assigned to either cognitive-behavioral rehabilitation or standard medical rehabilitation. Data were primarily collected via questionnaires at the beginning and end of the rehabilitation program, as well as three and twelve months after rehabilitation. For this analysis, only baseline, post-intervention, and 3-month follow-up data were used. Clinical examinations were conducted at the beginning and the end of rehabilitation.

### Participants

Patients aged 18 to 65 years who were approved for rehabilitation at the study center due to CVD (ICD-10 I05 to I71, I95, and I97) through the Federal German Pension Insurance or the German Pension Insurance North were included in the study. Additionally, eligibility required patients to have a diagnosed psychological comorbidity or indicate psychological distress, such as stress or exhaustion. This determination was made by the pension insurance, or the rehabilitation center based on the application documents prior to the commencement of rehabilitation. Patients with severe psychological disorders (schizophrenia, schizoaffective disorder, bipolar disorder, mania, severe unipolar depression), severely debilitating heart failure (at least NYHA Stage III), and insufficient proficiency in the German language were excluded.

### Variables

The selection of primary and secondary outcomes for the original trial was guided by a feasibility study [24] as well as proposed immediate performance measures in German cardiac rehabilitation [22, 25].

#### Physical activity

To assess the primary outcome, physical activity, participants were instructed to report up to three activities, specifying the type, frequency, and duration of each engagement over the past four weeks, preceding the respective study phase questionnaire [26]. The total weekly physical activity was calculated by multiplying the frequency and duration per activity, summing the minutes of each activity, and dividing the total by four.

#### Health-related variables

ESE was assessed using the German version of the Exercise Self-Efficacy Scale (ESES), which has demonstrated good test-retest reliability and internal consistency in measuring self-efficacy for physical activity [27, 28]. The questionnaire comprises ten statements (e.g., I am confident that I can achieve the activity and exercise goals I have set for myself), each rated on a 4-point scale ranging from “does not apply at all” to “applies completely”. The total score ranges from 10 to 40 points, with higher scores indicating greater confidence in maintaining regular physical activity. Additionally, motivation for a lifestyle change was assessed using a 5-point scale ranging from “certainly” (1 point) to “certainly not” (5 points). Participants who responded “certainly” or “rather yes” were categorized as motivated. Motivation for lifestyle changes served as a proxy indicator for patients’ intention to change health-related behaviors that are considered key cardiac risk factors, such as smoking, physical inactivity, and body weight. Furthermore, the smoking status was recorded.

The IRES-24 questionnaire was used to measure functional capacity by assessing difficulties encountered during physical activities, e.g., lifting heavy objects [29]. The extent of perceived difficulty for each activity was rated on a 5-point scale ranging from “impossible” to “without difficulty”. The total score ranges from 0 to 10 points, with higher scores indicating better functionality. Clinical examinations provided data on blood pressure, ergometer performance (watt/kg), as well as weight, and height, which were used to calculate the body mass index (BMI).

To assess general health, a visual analog scale (0-100 points) was used. Additionally, the 5-level EQ-5D measured health-related quality of life (HRQoL) across five dimensions: mobility, self-care, usual activities, pain/discomfort, and anxiety/depression. Each dimension had five response levels: no problems, slight problems, moderate problems, severe problems, and extreme problems. Health status combinations (e.g., 11111 for no problems in all dimensions) were converted into an index value reflecting the respective health status according to the preferences of the German general population, with 11,111 corresponding to a value of 1 [30].

#### Psychological variables

Indicators of psychological health, including depression, anxiety, and somatization, were measured using the Patient Health Questionnaire (PHQ). Depression (PHQ-9) and anxiety (GAD-7) were assessed with nine and seven items, respectively, using response options ranging from “not at all” (0 points) to “nearly every day” (3 points). PHQ-9 scores were categorized as none to minimal (0–4 points), low to moderate (5–14 points), and moderately severe to severe (15–27 points). GAD-7 scores were categorized as none to minimal (0–4 points), low to moderate (5–14 points), and severe (15–21 points). Somatization (PHQ-15) was evaluated with fifteen items rated as “not impaired”, “mildly impaired”, and “severely impaired”, with scores categorized as none to minimal (0–4 points), low to moderate (5–14 points), and severe (15–30 points) [31]. Cardiac anxiety was assessed using the 17-item German version of the Cardiac Anxiety Questionnaire (CAQ), with each item scored from 0 (“never”) to 4 (“always”) [32]. The total score was calculated as the average of all items (range 0–4 points). Higher scores on all psychological scales indicated greater severity.

#### Work participation

Self-rated work ability was assessed with the initial component of the Work Ability Index [33], which evaluates the current ability to work relative to the individual’s lifetime best. The score ranges from 0 (“completely unable to work”) to 10 (“work ability at its best”). Additional data on participants’ current job situations included employment status and the duration of sickness absence three months prior to rehabilitation.

#### Sociodemographic data

Data were collected on sex, age, native language, number of children, partnership status, highest educational degree, and vocational qualification. The educational level was determined according to the International Standard Classification of Education [34].

### Sample size calculation

A sample size of 286 participants was initially calculated for the randomized controlled trial [22]. A power analysis for this secondary analysis indicated that the sample was sufficient to detect a small correlation of 0.2 with at least 90% power, and was therefore appropriate for addressing our research questions.

### Statistical analysis

The sample characteristics were analyzed descriptively. To investigate the relationships between ESE and various potential explanatory variables, Pearson correlations were calculated for all continuous variables at baseline. The strength of the correlations was interpreted according to Dancey and Reidy [35], i.e. weak ranging from ± 0.1 to ± 0.3, moderate ranging from ± 0.4 to ± 0.6, or strong ranging from ± 0.7 to ± 0.9. The associations between ESE and nominal exploratory variables were assessed on baseline data using bivariate linear models. The strength of the associations was quantified using η² and interpreted according to Cohen, i.e. small if η² was at least 0.01, medium if η² was at least 0.06, or large if η² was at least 0.14 [36].

To examine whether ESE at the beginning of rehabilitation predicts the level of physical activity at the 3-month follow-up, a linear regression analysis was conducted. To assess whether ESE and physical activity have changed significantly over time, mean differences were calculated using paired t-tests. The relationship between changes in ESE from program initiation to completion and corresponding alterations in physical activity from baseline to the 3-month follow-up was assessed using a mixed-effects model. This approach included ESE as fixed effect, while accounting for individual variability through the inclusion of a random intercept.

In addition to the basic models, extended models were computed to control for potential confounders. For all regression models, the variable selection followed a standardized procedure. Initially, all variables that showed at least a moderate correlation with ESE at baseline were included in the respective models. A backward elimination approach was then applied to remove variables that did not contribute meaningfully to explaining the variance in the outcome. The elimination process was based on statistical significance, whereby variables with a p*-*value greater than 0.20 were sequentially excluded.

Both groups of the trial were analyzed as one cohort, as there were no differential changes in ESE or physical activity. The level of significance was set at *p* < 0.05. Missing values in multilevel scales were imputed by the individual means of the corresponding answered items, provided that the proportion of missing items did not exceed 25%. All assumptions for parametric testing were visually inspected using diagnostic plots and were found to be satisfied. All analyses were calculated with R version 4.4.1.

## Results

### Study sample

A total of 305 patients were enrolled in the CBR-CARDIO trial. This paper includes analyses based on data collected at three time points: baseline (*n* = 305), the end of rehabilitation (*n* = 284), and the 3-month follow-up (*n* = 262). Sample sizes for individual analyses varied depending on missing data: linear regression analyses included between 226 and 233 participants, while the mixed-effects model utilized up to 518 observations due to repeated measurements.

### Sample description

Table 1 provides an overview of the baseline sample characteristics. Participants initiated rehabilitation primarily due to hypertension (37%), ischemic heart disease (31,1%), or atrial fibrillation or other arrhythmias (14.2%). Out of a total of 233 psychological diagnoses, neurotic, stress-related, and somatoform disorders (ICD-10 F40–F48) were the most frequent, representing 45.5% of all cases. The second most frequent category was affective disorders, representing 24.9% of diagnoses, with all but one case classified as a form of depressive disorder (ICD-10 F32 & F33). Organic mental disorders (ICD-10 F10–F19) accounted for 17.2% of diagnoses, and behavioral syndromes associated with physiological disturbances and physical factors (ICD-10 F50–F59) made up 12.4%.

The average age was 56.4 years (SD = 6.95), with females accounting for 54.8% of the total population. At baseline, physical activity averaged 108 min per week (SD = 186), whereas confidence in maintaining physical activity reached a mean of 28.2 points (SD = 5.34). Approximately 85.2% of participants reported being non-smokers. Motivation for a lifestyle change was reported by 66.8%.

Among the sample, 18.0% of participants fell within the normal weight range (BMI 18.5–24.9) or less, 36.1% were classified as overweight (BMI 25.0–29.9), 30.5% fell into the category of obesity grade I (BMI 30.0–34.9), and 13.4% into obesity grade II/III (BMI ≥ 35.0). Functional capacity was rated at a mean of 5.84 points (SD = 2.11). The mean score for general health was 56.9 points (SD = 17.1), and HRQoL averaged a value of 0.728 (SD = 0.237).

Over 95% of participants were native German speakers, 43.3% had attained a high level of education, and 92.7% were employed. The mean self-rated work ability reached 5.34 points (SD = 2.23). Among those who reported sickness absence from work (65.1%), the average duration of absence amounted to 5.91 weeks (SD = 5.06). A total of 74.5% of participants were in stable partnerships, and 79.9% reported having at least one child.

Somatization scores varied among participants, with 7.5% reporting minimal symptoms, 56.2% experiencing low to moderate symptoms, and 36.3% indicating severe symptoms. The mean score for cardiac anxiety was 1.60 (SD = 0.673). With respect to depression, 18.8% of participants had no or minimal symptoms, 63.7% reported low to moderate symptoms, and 17.5% experienced moderately severe to severe symptoms. Anxiety levels were distributed as follows: 24.7% exhibited no or minimal symptoms, 62.9% had low to moderate symptoms, and 12.4% reported severe symptoms.

**Table 1:** Baseline characteristics

Characteristic	*N* = 305
Cardiovascular diseases	
Hypertension	113 (37.0%)
Ischemic heart disease	95 (31.1%)
Atrial fibrillation/other arrhythmias	43 (14.2%)
Other	54 (17.7%)
Physical activity (minutes), mean (SD)	108 (186)
Missing	21
Exercise self-efficacy, mean (SD)	28.2 (5.34)
Missing	19
Sex	
Male	138 (45.2%)
Female	167 (54.8%)
Age (years), mean (SD)	56.4 (6.95)
Weight (kg), mean (SD)	89.5 (18.4)
Smoking status^a^	
Yes	43 (14.8%)
No	248 (85.2%)
Missing	14
Motivation for lifestyle change ^a^	
Yes, or rather yes	193 (66.8%)
Uncertain, or less	96 (33.2%)
Missing	16
Partnership ^a^	
Yes	205 (74.5%)
No	70 (25.5%)
Missing	30
Number of children ^a^	
No children	58 (20.1%)
≤ 2 children	176 (61.1%)
≥ 3 children	54 (18.8%)
Missing	17
German mother tongue ^a^	
Yes	276 (95.5%)
No	13 (4.5%)
Missing	16
Education level ^a^	
Low or middle	165 (56.7%)
High	126 (43.3%)
Missing	14
Self-reported work ability, mean (SD)	5.34 (2.23)
Missing	18
Duration of sickness absence (weeks), mean (SD)	5.91 (5.06)
Missing	22
Employment status ^a^	
Employed	267 (92.7%)
Unemployed	21 (7.3%)
Missing	17
General health, mean (SD)	56.9 (17.1)
Missing	18
Functional capacity, mean (SD)	5.84 (2.11)
Missing	12
Somatization, mean (SD)	12.4 (5.29)
Missing	13
Cardiac anxiety, mean (SD)	1.60 (0.673)
Missing	14
HRQoL, mean (SD)	0.728 (0.237)
Missing	15
Depression, mean (SD)	9.47 (5.04)
Missing	13
Anxiety, mean (SD)	8.30 (4.78)
Missing	14

^a^Valid percentage values

The internal consistencies (Cronbach’s α) of the scales in the present sample ranged from 0.73 to 0.90, indicating acceptable to very good reliability. The primary scale, ESES, showed particularly high reliability (α = 0.90). Detailed values for all scales are presented in Supplementary Table 1.

### Factors associated with ESE

**Fig. 1:**
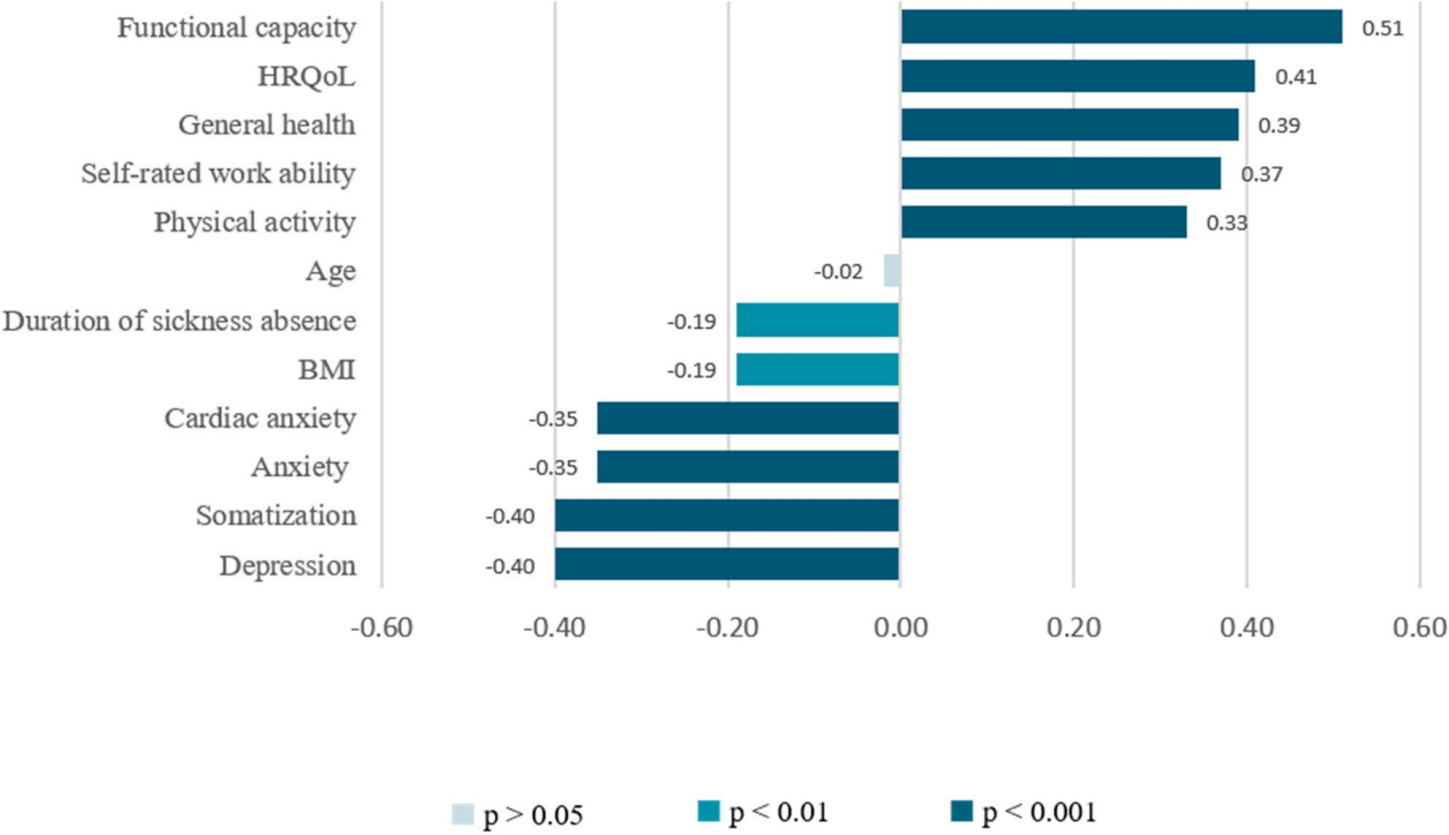
Pearson correlations between ESE and continuous variables at baseline

Correlation analysis conducted on baseline data (Fig. 1.) revealed a moderate correlation between ESE and functional capacity (*r* = 0.51, *p* < 0.001) and HRQoL (*r* = 0.41, *p* < 0.001). General health (*r* = 0.39, *p* < 0.001) and physical activity (*r* = 0.33, *p* < 0.001) showed weak positive associations with ESE, while the BMI was found to have a weak negative correlation with ESE (*r* = -0.19, *p* < 0.01). No correlation was identified between age and ESE (*r* = -0.02, *p* = 0.756).

All psychological factors showed significant associations with ESE. Depression (*r* = -0.44, *p* < 0.001) and somatization (*r* = -0.40, *p* < 0.001) demonstrated moderate negative correlations, while generalized anxiety (*r* = -0.35, *p* < 0.001) and cardiac anxiety (*r* = -0.35, *p* < 0.001) showed weak negative associations. Self-rated work ability showed a weak positive association with ESE (*r* = 0.37, *p* < 0.001), whereas the duration of sickness absence had a weak negative correlation with ESE (*r* = -0.19, *p* < 0.01).

The variance analysis revealed significant associations with small effect sizes (η²) between ESE and motivation for lifestyle change (η² = 0.04, *p* = 0.001), educational level (η² = 0.04, *p* = 0.001), employment status (η² = 0.02, *p* = 0.009), and sex (η² = 0.02, *p* = 0.018). No significant associations were found between ESE and native language, smoking status, number of children,

or partnership status.

### Prognostic relevance of ESE

To investigate whether the extent of ESE at the beginning of rehabilitation predicts activity behavior at the 3-month follow-up, a linear model was calculated, initially controlling only for baseline physical activity as a covariate (Table 2). In this context, ESE was identified as a significant predictor, with a regression coefficient of 0.10 (*p* = 0.006), indicating that each one-point increase in ESE corresponded to an increase in physical activity at follow-up by 0.10 h or approximately six minutes. Similarly, baseline physical activity was identified as a significant predictor of physical activity at follow-up (b = 0.29, *p* = < 0.001). For each hour increase in physical activity at baseline, physical activity at follow-up increased by 0.29 h, or roughly 17.4 min.

**Table 2:** Linear regression predicting physical activity at 3-month follow-up from baseline variables

Characteristic	Base model (*n* = 233)			Expanded model (*n* = 226)		
	b^a^	95% CI^b^	*p*-value	b^a^	95% CI^a^	*p*-value
ESE	0.10	0.03, 0.16	0.006*	0.08	0.00, 0.15	0.042*
Baseline physical activity	0.29	0.18, 0.40	< 0.001*	0.30	0.19, 0.41	< 0.001*
HRQoL	—	—	—	0.93	-0.68, 2.55	0.256

^a^ Unstandardized regression coefficient; ^b^ Confidence intervals; Base model R^2^ = 0.17; Expanded model R^2^ = 0.18; * *p* < .05

To account for potential confounders, all variables previously identified as moderately correlated with ESE and subjected to backward elimination were included in an expanded model. In this model, baseline ESE remained a significant predictor of follow-up physical activity with a slightly reduced regression coefficient of 0.08 (*p* = 0.042), corresponding to an increase of approximately five minutes per one-point increase in ESE. Baseline physical activity also remained a significant predictor (b = 0.30, *p* < 0.001), whereas HRQoL did not significantly predict follow-up activity (b = 0.93, *p* = 0.256).

### Changes in ESE and physical activity

Paired t-tests revealed a significant mean increase in ESE of 1.75 points (SD = 4.68, 95% CI: 1.19–2.31, *p* < 0.001) between baseline and the end of rehabilitation. Similarly, physical activity increased significantly, with a mean increase of 68.23 min (SD = 205.02, 95% CI: 42.04–94.52, *p* < 0.001) from baseline to the 3-month follow-up.

### Association between changes in ESE and changes in physical activity

To investigate the association between changes in ESE and physical activity over time, a mixed-effects model with random intercepts was employed (Table 3). The model revealed considerable individual variability in baseline activity, with random intercepts demonstrating a standard deviation of 111.5 min. When appropriately modeling this variability each one-point increase in ESE was associated with an average increase of 12.88 min in physical activity at the 3-month follow-up (*p* < 0.001). The combined fixed and random effects explained 45% of the variance in physical activity (conditional R² = 0.45).

In the expanded model (Table 3), changes in ESE (b = 8.80, *p* < 0.001) emerged as a significant predictor of changes in physical activity at follow-up. Specifically, each one-point increase in ESE was associated with an average increase of 8.80 min of physical activity at the 3-month follow-up. Moreover, changes in functional capacity (b = 11.34, *p* = 0.032) and depressive symptoms (b = − 5.19, *p* = 0.013) also significantly predicted changes in physical activity at follow-up (conditional R² = 0.53).

**Table 3:** Mixed-effects regression predicting changes in physical activity from changes in ESE over time

Characteristic	Base model (*n* = 518)^c^			Expanded model (*n* = 492)^c^		
	b^a^	95% CI^b^	*p*-value	b^a^	95% CI^b^	*p*-value
ESE	12.88	9.88, 15.89	< 0.001*	8.80	5.05, 12.45	< 0.001*
Functional capacity	—	—	—	11.34	1.04, 21.71	0.032*
Depression	—	—	—	-5.19	-9.25, -1.12	0.013*

^a^ Unstandardized regression coefficient; ^b^ Confidence interval, ^c^ Repeated measures; * *p* < .05

## Discussion

The correlation analyses revealed that, aside from age, all continuous health- and work-related, psychological, and sociodemographic factors were associated with ESE. Among categorical variables, small effect sizes were observed for the associations between ESE and motivation for lifestyle change, education level, employment status, and sex. Baseline ESE was found to be a significant predictor of physical activity at the 3-month follow-up. Paired t-tests revealed significant changes in both ESE and physical activity over time. The mixed-effects model, adjusted for individual heterogeneity, showed that improvements in ESE were significantly associated with increased activity levels at follow-up.

All methods employed to investigate the influence of ESE on physical activity consistently demonstrated a positive association. The mixed model specifically indicated that each one-point increase in ESE corresponded to approximately twelve additional minutes of weekly activity. Accordingly, an increase of one standard deviation in ESE (SD = 5.34) would be expected to result in roughly one additional hour of activity per week. Such an increase would be clinically meaningful in terms of activity duration, as it would account for around one-third of the World Health Organization’s (WHO) recommended 150 min of weekly activity for the primary and secondary prevention of CVD [37, 38]. However, exercise intensity is crucial, with the WHO recommending at least moderate-intensity activity, such as brisk walking or cycling. Given that our study did not differentiate between types of exercise, our ability to ascertain the full extent of clinically meaningful improvements in physical activity is limited.

Our findings are consistent with those of a systematic review, which found a positive relationship between ESE and initiating and maintaining exercise in patients with heart failure, particularly in the short term [39]. Another study on predictors of exercise adherence in home-based cardiac rehabilitation further confirmed that ESE is significantly associated with patients’ levels of activity [40]. This suggests that the observed association is likely, and ESE should be considered as an explanatory variable for the modification of physical activity in psychologically distressed patients undergoing cardiac rehabilitation.

The changes in ESE observed in this trial were comparable, yet higher than those reported in a recent study, in which patients undergoing cardiac rehabilitation showed a mean improvement of one point in ESE [41], compared to a mean increase of 1.75 points in our trial. However, published benchmarks of clinical importance for ESE are currently lacking. Establishing such benchmarks would be crucial for guiding future research [41].

Our analysis further confirmed an inverse association between depression and physical activity, consistent with previous findings in patients with CVD [42]. For instance, a recent longitudinal study in patients with peripheral artery disease reported that declines in physical activity preceded increases in depressive symptoms [18]. Furthermore, depression and CVD are closely interconnected through direct physiological mechanisms, each potentially worsening the other, and their co-occurrence is associated with poorer clinical outcomes, including higher risk of disability, hospitalization, and early mortality [43].

Given that physical activity has beneficial effects on both depressive symptoms and cardiovascular health, the routine implementation of cognitive-behavioral rehabilitation programs, such as the one evaluated in the CBR-CARDIO trial, appears warranted. Our findings indicate that these programs could be enhanced further by systematically raising awareness among healthcare professionals and by assessing ESE at the time of rehabilitation admission in order to identify at-risk patients and deliver individualized support. Targeted interventions to enhance ESE can build on core mechanisms of SCT, including graded mastery experiences (e.g., guided exercise practice), vicarious learning (e.g., peer role models), verbal encouragement, and reframing of physiological or emotional responses [19]. Such strategies, as demonstrated in previous self-efficacy enhancement programs [44], can be integrated into existing cardiac rehabilitation sessions, making them feasible and directly actionable.

However, behavior change is a complex process influenced by multiple interrelated factors in addition to ESE, with perceived benefits and barriers to exercise serving as key determinants of initiating and maintaining physical activity [45]. In this context, a lack of social support has been identified as a common barrier, with empirical evidence suggesting that married or partnered patients are approximately twice as likely to participate in cardiac rehabilitation compared to those who are single [46, 47]. Although our study did not identify partnership status as a predictor of either ESE or physical activity, addressing barriers such as insufficient social support may be crucial for enhancing ESE and sustaining higher activity levels. Cardiac telerehabilitation may also be a promising approach in overcoming participation and adherence barriers, as participants perceive it as an appropriate, convenient, and feasible alternative to center-based rehabilitation [48], potentially enhancing ESE further.

When interpreting the results, the following limitations should be considered. Firstly, as previously mentioned, the type of physical activity participants engaged in was disregarded in the analyses. This limits the interpretability of the findings, as research suggests that higher intensity levels of physical activity are associated with better health outcomes and decreased mortality in patients with CVD [49–51]. Secondly, the reliance on self-reported data may introduce potential biases. Research indicates that self-reported estimates of ESE tend to initially be overly optimistic [52, 53]. In some instances, these estimates might therefore even decrease following an exercise-based intervention, potentially leading to an underestimation of the associations analyzed. Physical activity was also assessed subjectively, which may introduce similar issues. A study examining the differences between subjectively and objectively measured physical activity in patients with coronary artery disease found that participants overestimated the amount of weekly moderate to vigorous physical activity they engaged in substantially [54]. Conversely, total physical activity may also be underestimated if work and household activities are not assessed, as was the case in our study [55]. Thirdly, given the relatively high scores of ESE at baseline, validity of the score may be compromised by a ceiling effect, potentially limiting its ability to detect further improvements in ESE beyond a certain point. In support of this, a recent study found that higher initial ESE scores were significantly linked to smaller changes in ESE at the end of cardiac rehabilitation [41]. Fourthly, the generalizability of the results may be limited for patients with a lower socioeconomic status, as over 90% of participants had no migration background, were employed, and had middle to high levels of education. Studies suggest that ethnic minorities and those with a lower socioeconomic status or education show less improvement in ESE and lower adherence to sustained exercise post-rehabilitation [15, 41, 56]. Finally, the analyses did not include 12-month follow-up data, which restricts our ability to assess long-term effects of ESE on physical activity among this population. Accordingly, a study by Slovinec D’Angelo et al. (2014) found that while ESE significantly predicted short-term exercise behavior following cardiac rehabilitation, it did not maintain its predictive power over the long term, a possibility that cannot be excluded in this study [57].

Despite the limitations, the study has several notable strengths. Firstly, the use of high-quality data from a randomized controlled trial, combined with a relatively large sample size, supports the internal validity of the findings. Secondly, the longitudinal design allows tracking of changes in physical activity over time and examination of temporal relationships, providing stronger evidence for potential causal effects. Finally, the focus on a psychologically vulnerable subgroup provides insights into a particularly distressed and underrepresented population.

In conclusion, our study suggests that ESE may have a considerable impact on behavior change in psychologically distressed patients undergoing cardiac rehabilitation. Therefore, it should be considered as an independent outcome parameter when designing intervention components in this setting. Routine screening and targeted enhancement of ESE in cardiac rehabilitation programs could help identify patients at risk for poor engagement in physical activity after rehabilitation and support more effective, personalized interventions.

## Supplementary Information

Supplementary Material 1.

## Data Availability

The datasets used and/or analyzed during the current study are available from the corresponding author on reasonable request.

## Abbreviations

CVD: Cardiovascular diseases
ESE: Exercise self-efficacy
HRQoL: Health-related quality of life
SCT: Social Cognitive Theory
WHO: World Health Organization
