# Supplementary material for: Influence of exercise self-efficacy on physical activity among psychologically distressed patients undergoing cardiac rehabilitation: secondary data analysis of a randomized controlled trial
Source: BMC Psychol. 2026 Feb 3;14:283. doi: 10.1186/s40359-026-04004-8 (PMC12951898; doi:10.1186/s40359-026-04004-8)
Supplement: Supplementary file 1 — Supplementary Material 1. [file 40359_2026_4004_MOESM1_ESM.docx]

**Supplementary Table 1**

| **Scale** | **Items** | **N** | **α*** |
| --- | --- | --- | --- |
| Functional capacity (IRES24) | 8 | 271 | 0.90 |
| Somatization (PHQ-15) | 13 | 250 | 0.78 |
| Cardiac anxiety (CAQ) | 17 | 260 | 0.88 |
| HRQoL (EQ-5D-5L) | 5 | 290 | 0.73 |
| Depression (PHQ-9) | 9 | 276 | 0.84 |
| General anxiety (GAD-7) | 7 | 285 | 0.88 |
| ESE (ESES) | 10 | 273 | 0.90 |

**^1^Cronbach’s alpha**
